# Three-Phase Fuel Deposition in a Long-Distance Migrant, the Red Knot (*Calidris canutus piersmai*), before the Flight to High Arctic Breeding Grounds

**DOI:** 10.1371/journal.pone.0062551

**Published:** 2013-04-30

**Authors:** Ning Hua, Theunis Piersma, Zhijun Ma

**Affiliations:** 1 Ministry of Education Key Laboratory for Biodiversity Science and Ecological Engineering, Institute of Biodiversity Science, Fudan University, Shanghai, China; 2 Animal Ecology Group, Centre for Ecological and Evolutionary Studies, University of Groningen, Groningen, The Netherlands; 3 Department of Marine Ecology, Royal Netherlands Institute for Sea Research, Den Burg, Texel, The Netherlands; Curtin University, Australia

## Abstract

Refuelling by migratory birds before take-off on long flights is generally considered a two-phase process, with protein accumulation preceding rapid fat deposition. The first phase expresses the demands for a large digestive system for nutrient storage after shrinkage during previous flights, the second phase the demands for fat stores to fuel the subsequent flight. At the last staging site in northward migration, this process may include expression of selection pressures both en route to and after arrival at the breeding grounds, which remains unascertained. Here we investigated changes in body composition during refuelling of High Arctic breeding red knots (*Calidris canutus piersmai*) in the northern Yellow Sea, before their flight to the tundra. These red knots followed a three-phase fuel deposition pattern, with protein being stored in the first and last phases, and fat being deposited mainly in the second phase. Thus, they did not shrink nutritional organs before take-off, and even showed hypertrophy of the nutritional organs. These suggest the build up of strategic protein stores before departure to cope with a protein shortage upon arrival on the breeding grounds. Further comparative studies are warranted to examine the degree to which the deposition of stores by migrant birds generally reflects a balance between concurrent and upcoming environmental selection pressures.

## Introduction

To survive the long-distance migrations between breeding and nonbreeding grounds, birds require large amounts of previously stored fuel in the form of fat and protein [1], [2]. Usually, long-distance migratory birds make one or more refuelling stops [3]. The High Arctic breeders among them will face additional pressures in their need to bring fuel stores to survive shortages upon arrival on the breeding grounds. For capital breeders, the fuel stores may also contribute to egg formation [4−8]. The short duration of the High Arctic summer confronts these migrants with the choice between the need to carry substantial stores to the Arctic and the need to minimize costs of refuelling, maintenance and transport [9].

The trade-offs between the different requirements of fuel deposition and consumption during staging and migratory flights in shorebirds relate to a considerable capacity to adjust the size of organs and stores, i.e. phenotypic flexibility [10], [11]. When storing fat (specialized deposits) and protein (mainly in the form of muscle) [12], [13] at staging sites, such birds mainly store protein in the early phase and fat in the later phase of a fuel deposition trajectory [13−15]. In the first phase, the organs consumed during the preceding flight are rebuilt, and in the second phase, the flight-related organs (such as flight muscles and heart) increase in mass while nutrition-related organs (such as gizzard and leg muscles) shrink before departure to decrease the burden for the next flight [13], [15], [16].

Whereas studies on fuel deposition patterns have focused on selection pressures experienced *en route* [13], [15], it is now realized that “upcoming” selection pressures on the breeding grounds may be important as well [17]. Because conditions on the tundra (with respect to weather and food and activity patterns) are so different from the coastal staging areas further south, and because Arctic summers are so short, comparison between fuel deposition patterns before different kinds of long flights (to different kinds of receiving areas) should inform us about the ways in which conditions in receiving areas influence organ dynamics during the previous storage phase [1], [18].

Here we analyse fuel storage in a subspecies of red knot (*Calidris canutus piersmai*) in the northern Yellow Sea, China, before a nonstop flight to breeding grounds in the New Siberian Arctic [19−21]. During northward migration, the knots make a nonstop flight of over 5000 km from the nonbreeding grounds in Australia to the staging sites along the Yellow Sea; after fuel deposition at these sites, they fly more than 4000 km to their breeding grounds [21], [22]. Departing from northern Yellow Sea to the breeding grounds in late May to commence breeding in early or mid-June [21], [22], the knots experience serious time constraints. Pressures *en route* and on the breeding grounds may jointly affect the fuel deposition during their final stopover. We discuss the patterns of temporal change in the organs and stores in a comparative context to identify selection pressures during migration.

## Materials and Methods

### Ethics Statement

Permission of fieldwork was granted by the Forest Department of Hebei Province. All work was approved by Institute of Biodiversity Science, Fudan University. Birds were handled and released soon after capture. Body feather sampling had little influence on the birds and majority of the sacrificed birds were euthanized after capture injury. All efforts were made to minimize the suffering and sacrifice.

### Experiment Protocol

Red knots were captured with clap nets [23] at Luannan (118.22°E, 39.07° N), in northern Bohai Bay, China, during their northward migration from 2008 to 2012. This is the largest known staging site in eastern Asia for red knots in the East Asian-Australasian Flyway, where at least 50% of the *piersmai* knots make a stop [22]. Birds were captured from early May to early June.

The *piersmai* knots were distinguished from the partially co-occurring *rogersi* knots on the basis of plumage colour and pattern [19], [22], [24]. Body mass was determined (to the nearest 0.1 g), and lengths of wing (to the nearest 1 mm), bill, head with bill, and tarsus were measured (to the nearest 0.1 mm) with stopped rulers and vernier calipers soon after birds were caught. We pulled 2–3 body feathers from each bird in 2008 and 2009. DNA was extracted from the pulp of feathers and molecular techniques were used to sex the birds [25].

A total of 32 carcasses were used in body composition analysis. The carcasses were individually sealed in airtight plastic bags and stored at −20°C before they were transported to the laboratory in Shanghai and dissected for body composition analysis. Before dissection, wing tracings were taken from 16 randomly selected individuals for the determination of wing area and aspect ratio; for the remaining birds, wing span was measured to determine wing area [26], [27]. The dissection procedure followed that in Royal Netherlands Institute for Sea Research (NIOZ) [2], [13]. The flight muscles (including *musculus pectoralis* and *musculus supracoracoideus*), leg muscles, gizzard, and other body parts were separated and weighed (±1 mg) with an analytical balance for the determination of fresh mass. The dry mass of each part was weighed after drying at 60°C to constant weight. Fat was extracted from each part in a Soxhlet apparatus using petroleum-ether (boiling point range 30–60°C) as the solvent, and the fat-free dry mass for each part was measured after drying again at 60°C. The total fat mass was calculated as the sum of fat mass extracted from all body parts.

### Data Analysis

**Figure 1:**
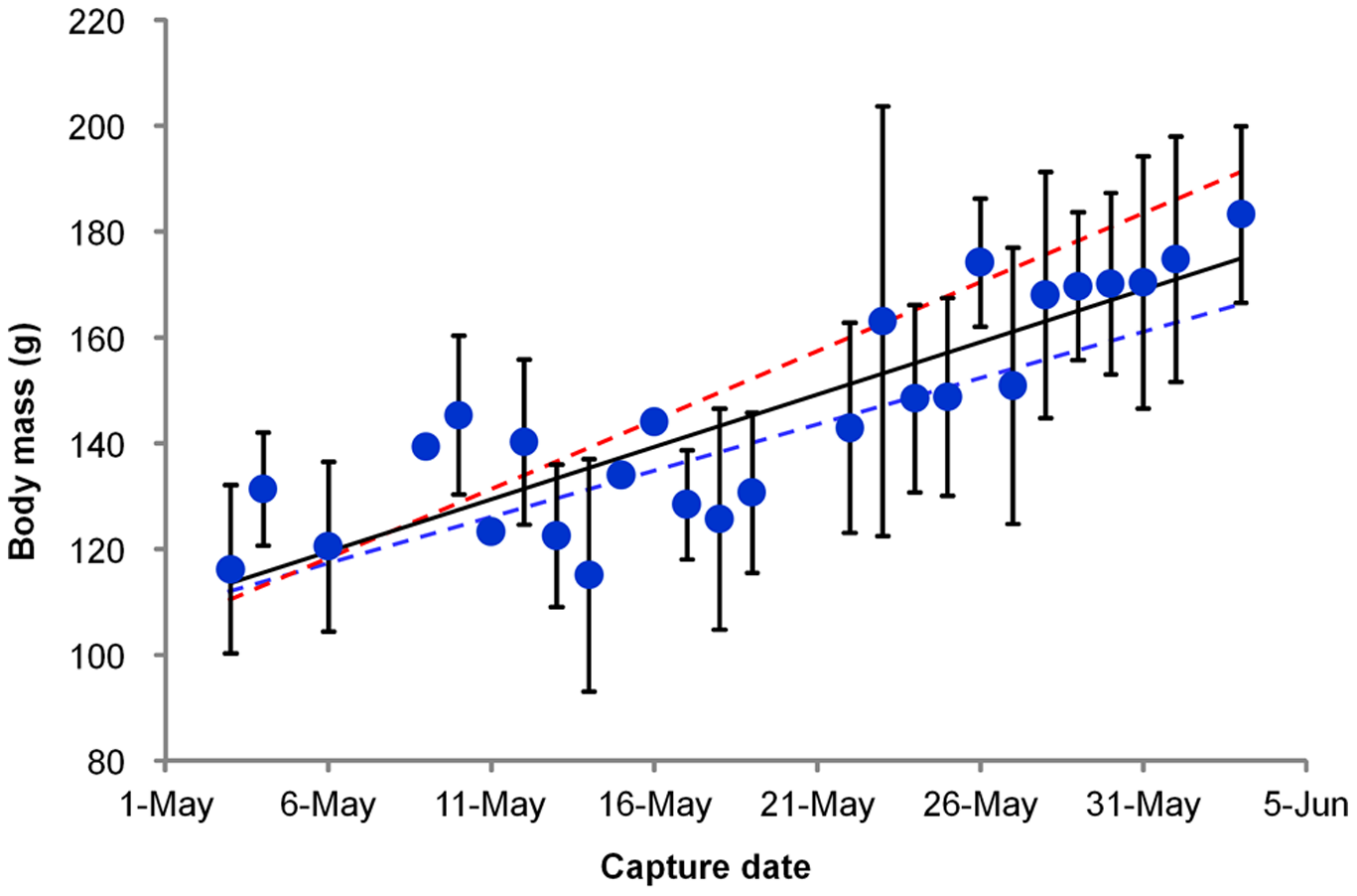
Body mass (mean ± SD) of red knots (*Calidris canutus piersmai*) captured on different dates. A total of 486 birds were sampled in 2008–2012 on the coast of the north Yellow Sea during northward migration. The solid line indicates least-square linear regression of total birds and the dashed lines indicate the linear regression of the females (red) and the males (blue).

Birds deposit both fat and protein (mainly in the form of muscle) during staging [13], [15], [28]. To understand the dynamics of fat and protein storage during staging, we used generalized linear models to evaluate the relationship between body composition (total fat mass, total lean dry mass (LDM), and LDM of flight muscles, gizzards, leg muscles, and “other nutrient organs”) and body mass. The first principle component of standardized LDM of liver, intestine, pancreas, and kidney was calculated to represent change of “other nutrient organs” as a whole. We used body mass rather than capture date as the independent variable for two reasons. First, changes in body composition are closely related to changes in body mass [12], [29]. Second, body mass greatly varied among individuals on the same day (Fig. 1), indicating lack of synchronization; body mass appears a better indicator of state than capture date. In addition to body mass, the models included body size (as the first principal component of structural body size measurements of wing length, head+bill length, and tarsus length) and sex as independent variables.

Second-order-corrected Akaike’s information criterion (AIC_c_) [30] was used to select the best models among the candidate models (see Table S1). Results indicated that body mass, and the square and cube of body mass, were always involved in the best models (except for the model of leg muscles, see Table S1). We therefore investigated the fuel deposition patterns as a function of body mass with piecewise linear models through the *Nonlinear Regression Analysis Program* (Advanced GUI version) [31].

The deviation between the average body mass of the heaviest five knots from the last dates and lightest five knots from the early dates that we captured was taken as “total body mass increase” during the entire refuelling phase [1]. We calculated the fuel composition of birds according to the piecewise fuel deposition models. The percentages of body mass gain represented by fat and LDM were then calculated.

We used *Flight* Program (Version 1.22) [27] to estimate the remaining fuel stores (fat and flight muscles) of knots arriving at the breeding grounds. This model gives conservative estimates for the birds because it does not take into account the environmental factors, such as wind assistance, leading to reduced flight costs. The flight distance from staging sites to breeding grounds was calculated by *Flight* as 4400 km for the birds according to their geographical coordinates [19]. The flight muscle mass was calculated as the fresh muscle mass minus the mass of fat extracted. The mass of indigested food in the alimentary tract mass was excluded from the total body mass because the birds’ guts would be emptied soon after take-off. The values of variables used in the simulation for the flight are listed in Table S2. The average body mass of birds captured during the last week of staging (after 25 May) was considered as the body mass of departing birds [22]. Birds with a body mass less than the estimated minimum body mass needed for a nonstop flight to the breeding grounds were excluded from calculations of the mean predicted body mass of departing birds. We also estimated body composition of the departing birds from our piecewise models. We further predicted the fuel stores of these birds on arrival at the breeding grounds using *Flight* Program [27].

## Results

**Figure 2:**
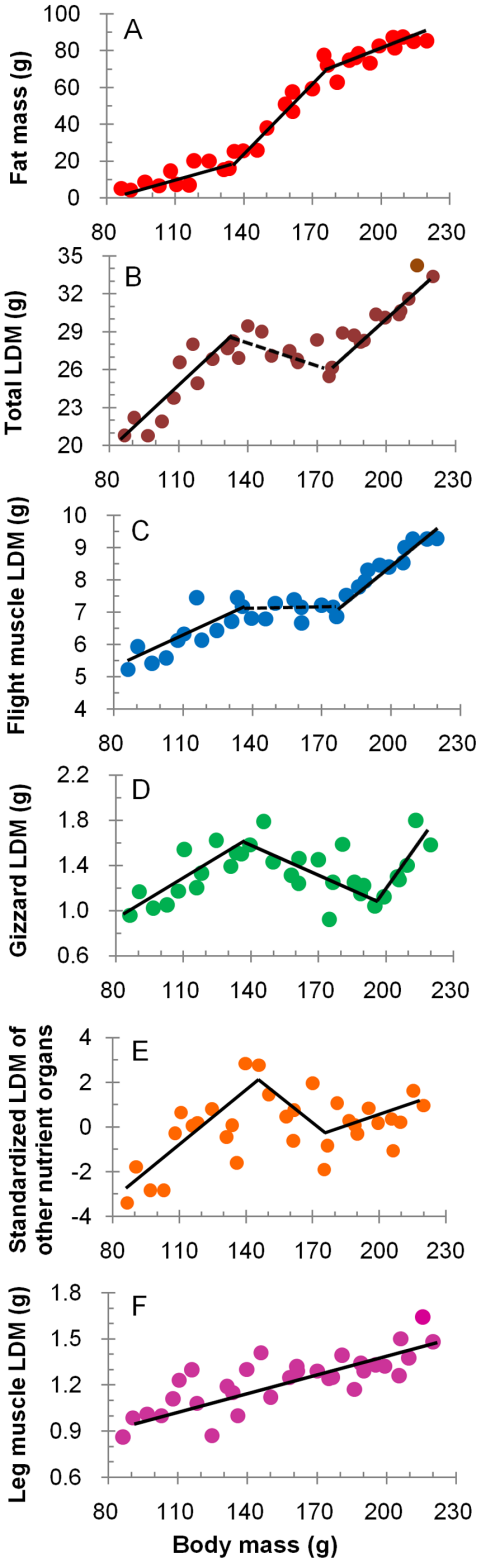
Regression analyses of the relationship between body components and body mass of *piersmai* red knots. Fuel deposition models are described with body mass as the independent variable and different body components as dependent variables, including fat mass (A), total lean dry mass (B), lean dry mass of flight muscles (C), gizzard (D), other nutrient organs (E) and leg muscles (F). Each point represents data from an individual bird. Significant regressions are presented with solid lines and insignificant regressions with dashed lines. The regression models were selected according to AIC_c_ in Table S1. The regression equations are listed in Table S3.

During their stage in the North Yellow Sea, the knots more than doubled their body mass, increasing from less than 100 g to more than 200 g (Fig. 1). The average body mass of the lightest five birds was 90.3±2.5 g (n = 5) and that of the heaviest five was 211.1±6.2 g (n = 5). Regression of body composition against body mass indicated that fuel deposition took place in three phases (Fig. 2A, 2B, Table S3). In the first phase (body mass<about 135 g), both fat and LDM increased steadily. In the second phase (body mass about 135–175 g), the rate of fat storage rapidly increased while LDM did not significantly change. In the third phase (body mass>about 175 g), the rate of fat storage slowed while the LDM increased again. Overall, the fat and protein accumulation were staggered, with protein storage occurring in the first and the third phase and rapid fat storage in the second phase (Fig. 2A, 2B, Table S3). Fat deposition contributed 34, 100, and 40% to the increase in body mass in the first, second, and third phase, respectively. Across the entire refueling period, fat mass increased from 4.8 to 84.8 g and fat deposition contributed 66.2% to the increase in body mass, while LDM increased from 21.2 to 32.2 g and dry protein deposition contributed 9.1% to the increase in body mass, the rest being the water that comes with dry protein.

The LDM of flight muscles changed in proportion to total LDM, the three phases again being evident (Fig. 2C, Table S3). Gizzards also showed a three-phase change (Fig. 2D, Table S3). Although gizzard LDM decreased in the second phase, it increased again in the third phase and attained the highest value when body mass reached the maximum. The average standardized LDM of other nutrition-related organs changed in a similar three-phase way as gizzard LDM (Fig. 2E, Table S3). In contrast, the LDM of leg muscles increased throughout the staging period (Fig. 2F).

There were sexual differences in fuel deposition of the knots. The body mass of departing females (captured after 25 May, 187.1±14.9 g, n = 38) was significantly higher than that of the males (172.8±7.6 g, n = 36) (t = 5.22, df = 72, *P*<0.001, Fig. 1). Females deposited more fat (males: 67.4 g, females: 75.2 g) and more protein (males: 26.6 g LDM, females: 28.3 g LDM) than males. Regression analysis also indicated sex differences in fat and flight muscle mass changes (sex remained as a factor in the best model) and lean dry mass change (sex was kept in the alternative model). There was no significant difference in the nutrient organ masses between the males and the females during fuel deposition (Table S1).

**Figure 3:**
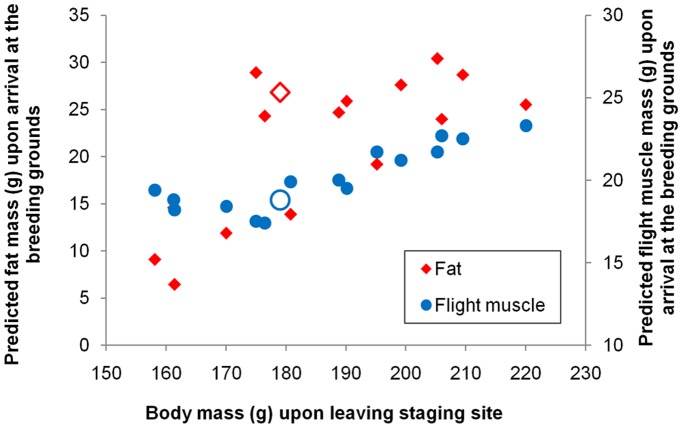
Prediction of fat and flight muscle mass of *piersmai* red knots upon arrival at breeding grounds. Fat and protein consumptions during flight were simulated according to *Flight* Program. Only birds with sufficient mass (body mass >158 g) for arrival at the breeding grounds were analyzed. The open symbols indicate a bird with a body mass of 178.7 g, which was the average body mass of birds captured during the last week of staging.

Modelling the fuel consumption of the knots during migratory flight indicated that birds would be able to make it to the breeding grounds on the New Siberian Islands if they would achieve departure body mass values above 158 g (Fig. 3). 75% of the knots we captured during the last week of staging (after 25 May) weighed over 158 g, and the average body mass was 178.7±11.3 g (n = 189). This departing weight would lead to a residual body mass of 109 g at the breeding grounds, with a fat content of 23.3 g or 21.4% of total body mass and fresh flight muscle mass of 18.7 g or 17.2% of total body mass (Fig. 3).

## Discussion

Unlike previously observed refuelling patterns of long-distance migratory birds [13], [14], [32], the knots displayed a three-phase refuelling in which additional protein was stored in the third phase. The increase of LDM in the first refuelling phase could reflect the restoration of organs and tissues consumed during the long-distance migratory flight from nonbreeding to staging sites [2], [13]. This restoration of organs and tissues is a prerequisite to rapid fat deposition in the second phase. Because the maintenance of metabolically active tissues consumes fuel [33], [34], the hypertrophy of muscles and other organs before take-off would decrease the self-maintenance costs during stopover. The LDM for the flight muscles, which is the major component of protein in birds, remains stable until the last fueling phase, when it increases rapidly. The staggering of fat and protein deposition during these refuelling phases suggests a conflict between rapid deposition of different fuel types.

The knots deposited large amounts of fuel at the last staging site in the north Yellow Sea during the migration to the breeding grounds. During the complete refuelling episode, their lean dry mass increased 51.8% and fat mass increased 16.7 times from the initial values at arrival. Although overload of fuel at staging sites has been reported before in the Arctic breeders [4], [35], [36], the fuel deposition, especially the protein deposition, of *piersmai* knots is greater than in similar stages in other birds [1]. Modelling of the flight fuel consumption indicates that most birds stored much more than needed for a nonstop flight to the breeding grounds. Consequently, the knot at least partially exhibits a capital breeding strategy, enabling reproduction to partially depend on energy and nutrient stores [36]. On the breeding grounds, requirements differ between the sexes as only the females need to produce the fat and protein that goes into the four eggs. This may explain why female knots deposited more protein before take-off than males.

Previous studies have indicated that birds shrink digestive organs and leg muscles before non-stop and long-distance flights for the presumed purpose of decreasing the mass burden [13], [15], [37]. The knots, who will make a non-stop flight of over 4000 km after staging at the north Yellow Sea, however, rather showed hypertrophy of the nutrient organs before departure along with the gains in leg musculature. This suggests that upon arrival on the tundra this protein, and/or the organs in which the protein is stored, are needed. In addition, arriving with suitably sized organs and tissues reduces the requirement for physical restoration [17]. Thus, in this case the benefits of maintaining internal organs may outweigh the benefits of a decrease in flight cost. Note that the gizzard of *piersmai* knots (4.59±0.84 g, n = 32, fresh mass) is smaller than that at other staging sites (7.95–8.17 g) [38], reflecting the high quality of prey encountered in northern Bohai Bay (Yang et al. MS).

Whereas most previous studies reporting fuel deposition and phenotypic flexibility of long-distance migratory birds have emphasized the function of *en route* selection pressures [13], [15], the three-phase fuel deposition and the hypertrophy of the nutrition-related organs pre-departure suggests that compositional changes on staging areas reflect selection to both the ensuing flights and the conditions and requirements at the next destination. In the present case there is evidence for a seasonal carry-over effect [39], emphasizing that different phases in the life cycle of migratory birds are intimately connected [18], [40], [41].

## Supporting Information

Table S1 **Regression models for predicting fuel deposition of** ***piersmai*** **red knots at final staging sites in the north Yellow Sea during northward migration.** Dependent variables were fat mass, total lean dry mass, lean dry mass of flight muscles, gizzard, leg muscles, standardized lean dry mass of other nutrient organs and fresh mass of flight muscles. Models were compared with the second-order-corrected Akaike’s information criterion (AIC_c_, Burnham and Anderson 2002). M = body mass (g), G = gender (male vs. female, dummy coded, male = 1 and female = 0), S = structural size (first principal components of wing length, head+bill length, and Tarsus length), *K* = number of estimable parameters, *Wi* = model weight. Models are ranked according to the ascending sequence of AIC_c_ values. Only the first ten models are listed for each prediction. For the models with Delta AIC_c_ ≤2, the model parameters for the selected independent variables are listed in parentheses with (+) indicating positive and (-) negative correlation.
(DOC)

Table S2 **Values for variables (following Pennycuick and Battley 2003, Pennycuick 2008) used in the simulation for the flight of red knots.** References:Pennycuick C J, Battley P (2003) Burning the engine: a time-marching computation of fat and protein consumption in a 5420-km non-stop flight by great knots, *Calidris tenuirostris*. Oikos 103∶323–332. Pennycuick C J (2008) Modelling the Flying Bird. London: Academic Press. 216 p.
(DOC)

Table S3 **Regression analyses of the relationship between body components (Y) and body mass (X) of** ***piersmai*** **red knots.**The regression models were selected according to AIC_c_ in Table S1. The piecewise regression equations and breakpoints for each equation were calculated through the Nonlinear Regression Analysis Program (Advanced GUI version).
(DOC)
