# Supplementary material for: Three-Phase Fuel Deposition in a Long-Distance Migrant, the Red Knot (Calidris canutus piersmai), before the Flight to High Arctic Breeding Grounds
Source: PLoS One. 2013 Apr 30;8(4):e62551. doi: 10.1371/journal.pone.0062551 (PMC3640059; doi:10.1371/journal.pone.0062551)
Supplement: Table S1 — Regression models for predicting fuel deposition of piersmai red knots at final staging sites in the north Yellow Sea during northward migration. Dependent variables were fat mass, total lean dry mass, lean dry mass of flight muscles, gizzard, leg muscles, standardized lean dry mass of other nutrient organs and fresh mass of flight muscles. Models were compared with the second-order-corrected Akaike’s information criterion (AICc, Burnham and Anderson 2002). M = body mass (g), G = gender (male vs. female, dummy coded, male = 1 and female = 0), S = structural size (first principal components of wing length, head+bill length, and Tarsus length), K = number of estimable parameters, Wi = model weight. Models are ranked according to the ascending sequence of AICc values. Only the first ten models are listed for each prediction. For the models with Delta AICc ≤2, the model parameters for the selected independent variables are listed in parentheses with (+) indicating positive and (-) negative correlation. (DOC) [file pone.0062551.s001.doc]

Table S1. Regression models for predicting fuel deposition of *piersmai* red knots at final staging sites in the north Yellow Sea during northward migration.

| Model | Independent variables | | *K* | AIC_c_ | Delta AIC_c_ | | *Wi* | R^2^ | *p* |
| --- | --- | --- | --- | --- | --- | --- | --- | --- | --- |
| **Fat mass** | |  | | | |  | | | |
| 1 | M (-6.40), M^2^ (0.05), M^3^ (-1.00×10^-4^), G (4.32) | | 5 | 204.22 | 0 | | 0.44 | 0.98 | < 0.001 |
| 2 | M (-6.83), M^2^ (0.05), M^3^ (-1.08×10^-4^) | | 4 | 204.73 | 0.51 | | 0.34 | 0.97 | < 0.001 |
| 3 | M, M^2^, M^3^, S | | 5 | 206.65 | 2.44 | | 0.13 | 0.98 | < 0.001 |
| 4 | M, M^2^, M^3^, G, S | | 6 | 207.34 | 3.13 | | 0.09 | 0.98 | < 0.001 |
| 5 | M^2^, M^3^, G | | 4 | 222.79 | 18.58 | | < 0.01 | 0.96 | < 0.001 |
| 6 | M^2^, G | | 3 | 223.11 | 18.90 | | < 0.01 | 0.95 | < 0.001 |
| 7 | M^2^, M^3^ | | 3 | 224.46 | 20.25 | | < 0.01 | 0.95 | < 0.001 |
| 8 | M, M^2^, G | | 4 | 224.74 | 20.53 | | < 0.01 | 0.95 | < 0.001 |
| 9 | M, G | | 3 | 224.99 | 20.77 | | < 0.01 | 0.95 | < 0.001 |
| 10 | M^2^, G, S | | 4 | 225.24 | 21.02 | | < 0.01 | 0.95 | < 0.001 |
| **Lean dry mass** | |  | | | |  | | | |
| 1 | M (1.86), M^2^(-0.01), M^3^(2.61×10^-5^) | | 4 | 113.64 | 0 | | 0.58 | 0.86 | < 0.001 |
| 2 | M(1.80), M^2^(-0.01), M^3^(2.50×10^-5^), G (-0.64) | | 5 | 115.39 | 1.75 | | 0.24 | 0.87 | < 0.001 |
| 3 | M, M^2^, M^3^, S | | 5 | 116.55 | 2.91 | | 0.13 | 0.86 | < 0.001 |
| 4 | M, M^2^, M^3^, G, S | | 6 | 118.69 | 5.06 | | 0.05 | 0.87 | < 0.001 |
| 5 | M | | 2 | 133.78 | 20.14 | | < 0.01 | 0.70 | < 0.001 |
| 6 | M, G | | 3 | 134.02 | 20.39 | | < 0.01 | 0.72 | < 0.001 |
| 7 | M^2^ | | 2 | 135.43 | 21.79 | | < 0.01 | 0.68 | < 0.001 |
| 8 | M, S | | 3 | 135.86 | 22.22 | | < 0.01 | 0.70 | < 0.001 |
| 9 | M, M^2^, G | | 4 | 135.93 | 22.29 | | < 0.01 | 0.73 | < 0.001 |
| 10 | M, M^2^ | | 3 | 136.25 | 22.61 | | < 0.01 | 0.70 | < 0.001 |
| **Flight muscle mass** | |  | | | |  | | | |
| 1 | M (0.26), M^2^ (-1.69×10^-3^), M^3^(3.79×10^-6^), G (-0.42) | | 5 | 31.68 | 0 | | 0.49 | 0.92 | < 0.001 |
| 2 | M, M^2^, M^3^, G, S | | 6 | 34.07 | 2.40 | | 0.15 | 0.92 | < 0.001 |
| 3 | M^2^, G | | 3 | 35.63 | 3.95 | | 0.07 | 0.89 | < 0.001 |
| 4 | M, M^2^, M^3^ | | 4 | 35.64 | 3.96 | | 0.07 | 0.90 | < 0.001 |
| 5 | M, G | | 3 | 35.96 | 4.28 | | 0.06 | 0.89 | < 0.001 |
| 6 | M, M^3^, G | | 4 | 37.62 | 5.94 | | 0.03 | 0.89 | < 0.001 |
| 7 | M^3^, G | | 3 | 37.87 | 6.19 | | 0.02 | 0.88 | < 0.001 |
| 8 | M^2^, G, S | | 4 | 37.97 | 6.29 | | 0.02 | 0.89 | < 0.001 |
| 9 | M, M^2^, G | | 4 | 38.06 | 6.38 | | 0.02 | 0.89 | < 0.001 |
| 10 | M^2^, M^3^, G | | 4 | 38.44 | 6.76 | | 0.02 | 0.89 | < 0.001 |
| **Gizzard mass** | |  | | | |  | | | |
| 1 | M (0.19), M^2^ (-1.27×10^-3^), M^3^ (2.68×10^-6^) | | 4 | -12.81 | 0 | | 0.66 | 0.46 | < 0.001 |
| 2 | M, M^2^, M^3^, G | | 5 | -9.93 | 2.88 | | 0.16 | 0.46 | 0.002 |
| 3 | M, M^2^, M^3^, S | | 5 | -9.77 | 3.05 | | 0.14 | 0.46 | 0.002 |
| 4 | M, M^2^, M^3^, G, S | | 6 | -6.69 | 6.12 | | 0.03 | 0.46 | 0.005 |
| 5 | - | | 1 | -1.20 | 11.62 | | < 0.01 | - | - |
| 6 | M | | 2 | 0.08 | 12.89 | | < 0.01 | 0.04 | 0.299 |
| 7 | M^2^ | | 2 | 0.41 | 13.23 | | < 0.01 | 0.03 | 0.381 |
| 8 | M^3^ | | 2 | 0.59 | 13.41 | | < 0.01 | 0.02 | 0.437 |
| 9 | M, M^2^ | | 3 | 0.60 | 13.41 | | < 0.01 | 0.10 | 0.227 |
| 10 | S | | 2 | 0.78 | 13.59 | | < 0.01 | 0.01 | 0.511 |
| **Leg muscle mass** | |  | | | |  | | | |
| 1 | M (3.51×10^-3^) | | 2 | -44.16 | 0 | | 0.20 | 0.61 | < 0.001 |
| 2 | M^2^ (1.12×10^-5^) | | 2 | -43.31 | 0.85 | | 0.13 | 0.60 | < 0.001 |
| 3 | M^3^ | | 2 | -41.92 | 2.24 | | 0.07 | 0.59 | < 0.001 |
| 4 | M, M^2^, M^3^ | | 4 | -41.92 | 2.25 | | 0.07 | 0.65 | < 0.001 |
| 5 | M, G | | 3 | -41.78 | 2.38 | | 0.06 | 0.62 | < 0.001 |
| 6 | M, S | | 3 | -41.56 | 2.60 | | 0.05 | 0.62 | < 0.001 |
| 7 | M, M^2^ | | 3 | -41.56 | 2.61 | | 0.05 | 0.62 | < 0.001 |
| 8 | M, M^3^ | | 3 | -41.54 | 2.62 | | 0.05 | 0.61 | < 0.001 |
| 9 | M^2^, M^3^ | | 3 | -41.09 | 3.07 | | 0.04 | 0.61 | < 0.001 |
| 10 | M^2^, S | | 3 | -40.83 | 3.34 | | 0.04 | 0.61 | < 0.001 |
| **Other nutrient organ mass** | |  | | | |  | | | |
| 1 | M (1.00), M^2^(-6.19×10^-3^), M^3^(1.25×10^-5^) | | 4 | 109.17 | 0 | | 0.59 | 0.47 | < 0.001 |
| 2 | M, M^2^, M^3^, G | | 5 | 111.99 | 2.82 | | 0.14 | 0.47 | 0.001 |
| 3 | M, M^2^, M^3^, S | | 5 | 112.07 | 2.90 | | 0.14 | 0.47 | 0.001 |
| 4 | M, M^2^ | | 3 | 115.23 | 6.06 | | 0.03 | 0.30 | 0.006 |
| 5 | M, M^2^, M^3^, G, S | | 6 | 115.25 | 6.07 | | 0.03 | 0.47 | 0.003 |
| 6 | M, M^3^ | | 3 | 116.38 | 7.21 | | 0.02 | 0.27 | 0.010 |
| 7 | M^2^, M^3^ | | 3 | 117.82 | 8.65 | | 0.01 | 0.24 | 0.018 |
| 8 | M, M^2^, G | | 4 | 117.99 | 8.82 | | 0.01 | 0.30 | 0.017 |
| 9 | M, M^2^, S | | 4 | 118.04 | 8.87 | | 0.01 | 0.30 | 0.017 |
| 10 | M, M^3^, G | | 4 | 119.16 | 9.99 | | < 0.01 | 0.28 | 0.027 |

Dependent variables were fat mass, total lean dry mass, lean dry mass of flight muscles, gizzard, leg muscles, standardized lean dry mass of other nutrient organs and fresh mass of flight muscles. Models were compared with the second-order-corrected Akaike’s information criterion (AIC_c_, Burnham and Anderson 2002). M = body mass (g), G = gender (male vs. female, dummy coded, male = 1 and female = 0), S = structural size (first principal components of wing length, head + bill length, and Tarsus length), *K* = number of estimable parameters, *Wi* = model weight. Models are ranked according to the ascending sequence of AIC_c_ values. Only the first ten models are listed for each prediction. For the models with Delta AIC_c_ ≤ 2, the model parameters for the selected independent variables are listed in parentheses with (+) indicating positive and (-) negative correlation.
