# Supplementary material for: Three-Phase Fuel Deposition in a Long-Distance Migrant, the Red Knot (Calidris canutus piersmai), before the Flight to High Arctic Breeding Grounds
Source: PLoS One. 2013 Apr 30;8(4):e62551. doi: 10.1371/journal.pone.0062551 (PMC3640059; doi:10.1371/journal.pone.0062551)
Supplement: Table S2 — Values for variables (following Pennycuick and Battley 2003, Pennycuick 2008) used in the simulation for the flight of red knots. References:Pennycuick C J, Battley P (2003) Burning the engine: a time-marching computation of fat and protein consumption in a 5420-km non-stop flight by great knots, Calidris tenuirostris. Oikos 103∶323–332. Pennycuick C J (2008) Modelling the Flying Bird. London: Academic Press. 216 p. (DOC) [file pone.0062551.s002.doc]

Table S2. Values for variables (following Pennycuick and Battley 2003, Pennycuick 2008) used in the simulation for the flight of red knots.

| Variables | Values |
| --- | --- |
| Body drag coefficient | 0.1 |
| Induced power factor | 1.2 |
| Profile power constant | 8.4 |
| Air density (kg/m^3^) | 0.909 |
| Altitude (m asl) | 3000 |
| Gravity (m/s^2^) | 9.81 |
| Fat energy density (J/kg) | 3.90E+07 |
| Dry protein energy density (J/kg) | 1.83E+07 |
| Protein hydration ratio | 2.2 |
| Conversion efficiency | 0.23 |
| Respiration factor | 1.1 |
| Mitochondria inverse power density (m^3^/W) | 1.20E-06 |

References:

Pennycuick C J, Battley P (2003) Burning the engine: a time-marching computation of fat and protein consumption in a 5420-km non-stop flight by great knots, *Calidris tenuirostris*. Oikos 103: 323–332.

Pennycuick C J (2008) Modelling the Flying Bird. London: Academic Press. 216 p.
