# Supplementary material for: Three-Phase Fuel Deposition in a Long-Distance Migrant, the Red Knot (Calidris canutus piersmai), before the Flight to High Arctic Breeding Grounds
Source: PLoS One. 2013 Apr 30;8(4):e62551. doi: 10.1371/journal.pone.0062551 (PMC3640059; doi:10.1371/journal.pone.0062551)
Supplement: Table S3 — Regression analyses of the relationship between body components (Y) and body mass (X) of piersmai red knots.The regression models were selected according to AICc in Table S1. The piecewise regression equations and breakpoints for each equation were calculated through the Nonlinear Regression Analysis Program (Advanced GUI version). (DOC) [file pone.0062551.s003.doc]

Table S3. Regression analyses of the relationship between body components (Y) and body mass (X) of *piersmai* red knots.

| **Regression equation** | | | | **ANOVA** | | | |
| --- | --- | --- | --- | --- | --- | --- | --- |
| Body component | Intercept | Body mass (g) at breakpoint | Slope | F | *p* | R^2^ | N |
| Fat mass (g) | −25.63 (*p* = 0.011) | X_1_ = 138 (*p* < 0.001 )  X_2_ = 175 (*p* < 0.001) | K_1_ = 0.34 (for X ≤ 138, *p* < 0.001)  K_2_ = 1.34 (for 138 < X ≤ 175, *p* < 0.001)  K_3_ = 0.40 (for X > 175, *p* < 0.001) | 281.30 | < 0.001 | 0.98 | 32 |
| Total LDM (g) | 5.68 (*p* = 0.06) | X_1_ = 132 (*p* < 0.001 )  X_2_ = 176 (*p* < 0.001) | K_1_ = 0.17 (for X ≤ 132, *p* < 0.001)  K_2_ = −0.045 (for 132 < X ≤ 176, *p* = 0.10)  K_3_ = 0.16 (for X > 176, *p* < 0.001) | 43.60 | < 0.001 | 0.89 | 32 |
| Flight muscle LDM (g) | 2.25 (*p* < 0.001) | X_1_ = 134 (*p* < 0.001 )  X_2_ = 174 (*p* < 0.001) | K_1_ = 0.036 (for X ≤ 134, *p* < 0.001)  K_2_ = 0.0010 (for 134 < X ≤ 174, *p* = 0.93)  K_3_ = 0.053 (for X > 174, *p* < 0.001) | 63.21 | < 0.001 | 0.92 | 32 |
| Gizzard LDM (g) | 0.059 (*p* = 0.84) | X_1_ = 141 (*p* < 0.001 )  X_2_ = 198 (*p* < 0.001) | K_1_ = 0.011 (for X ≤ 141, *p* < 0.001)  K_2_ = −0.0088 (for 141 < X ≤ 198, *p* = 0.005)  K_3_ = 0.028 (for X > 198, *p* =0.007) | 7.62 | < 0.001 | 0.59 | 32 |
| Standardized LDM of other nutrient organs | −9.67 (*p* < 0.001) | X_1_ = 141 (*p* < 0.001 )  X_2_ = 175 (*p* < 6.001) | K_1_ = 0.079 (for X ≤ 146, *p* < 0.001)  K_2_ = −0.083 (for 141 < X ≤ 175, *p* < 0.001)  K_3_ = 0.031 (for X > 175, *p* < 0.001) | 5.55 | 0.001 | 0.52 | 32 |
| Leg muscle LDM (g) | 0.68 (*p* < 0.001) | NA | K = 0.0035 (*p* < 0.001) | 47.88 | < 0.001 | 0.61 | 32 |

The regression models were selected according to AIC_c_ in Table S1. The piecewise regression equations and breakpoints for each equation were calculated through the *Nonlinear Regression Analysis Program* (Advanced GUI version).
